# Global Population Trends and Human Use Patterns of *Manta* and *Mobula* Rays

**DOI:** 10.1371/journal.pone.0074835

**Published:** 2013-09-11

**Authors:** Christine A. Ward-Paige, Brendal Davis, Boris Worm

**Affiliations:** Biology Department, Dalhousie University, Halifax, Nova Scotia, Canada; Aristotle University of Thessaloniki, Greece

## Abstract

Despite being the world’s largest rays and providing significant revenue through dive tourism, little is known about the population status, exploitation and trade volume of the Mobulidae (mobulids; *Manta* and *Mobula* spp.). There is anecdotal evidence, however, that mobulid populations are declining, largely due to the recent emergence of a widespread trade for their gill rakers, which is reflected in increasing Food and Agriculture Organization landings trends. Here, we present results from two dedicated diver surveys, one from the eManta project, which includes summary observations from ninety 10°x10° regions with ∼200–62,000 dives per region, and the other from the Reef Environmental Education Foundation, which includes spatially more detailed observations from 3 regions with ∼4,000–118,000 dives per region. We show that mobulids as a group, which includes eleven species, have globally and regionally restricted distributions, typically have low sighting frequency (<1% of dives) and aggregate in only a few locations. Of the regions surveyed by divers, almost half (47%) report declining mobulid sightings over the last decade. Divers indicate that although mobulid ecotourism occurs in many regions (45% of those reported, n = 41) they are considered protected in only 32% of the regions. Mobulids being fished or sold in local markets were reported from 16% and 12% of regions, respectively, with most being adjacent to mobulid abundance hotspot and ecotourism regions (e.g. Sri Lanka, Indonesia, east Africa). Identification of regions where ecotourism and exploitation are at odds could help prioritize conservation efforts. Vulnerability analysis, using life history characteristics, indicates that *Manta* spp. are vulnerable to exploitation, tolerating only low fishing mortality rates; data limitations prohibited such analysis for *Mobula* spp. Our analyses support previous studies in showing the need for improved conservation and monitoring efforts, and suggest that international and enforceable management policies are required to prevent further population decline.

## Introduction

**Figure 1:**
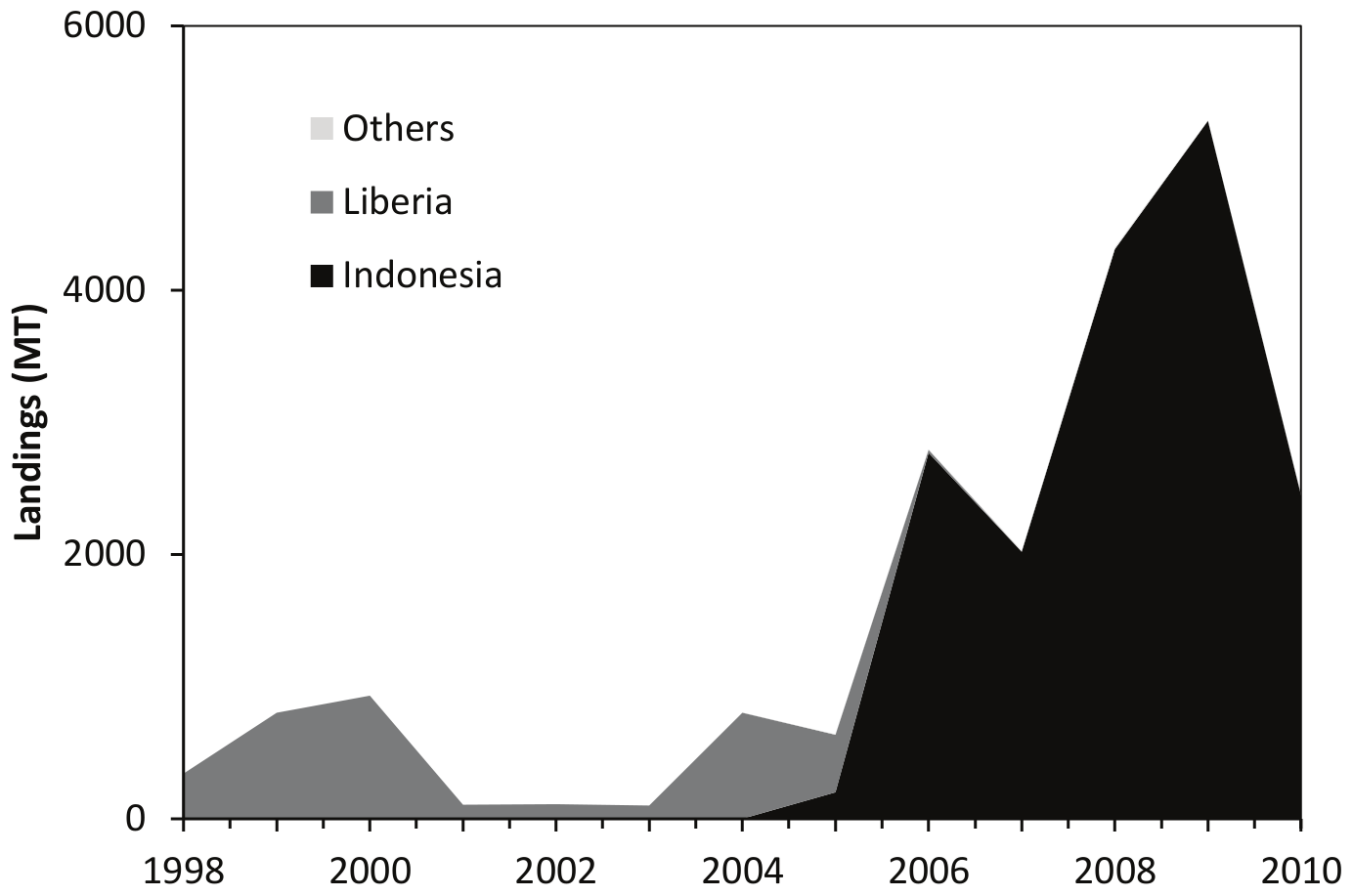
Landings trends. Globally reported *Manta* and *Mobula* landings were derived from the FAO Fisheries and Aquaculture FishStatJ dataset [36] by country (in metric tons). The relevant FAO species categories (“Giant manta” and “manta/devil rays, nei”) were pooled. An undifferentiated category of ‘rays, stingrays, manta nei’, was excluded from our dataset, because the proportion of mobulids in this category could not be estimated. No mobulid landings were reported prior to 1998. Note that other countries’ (Spain and Ecuador) reported landings were relatively small (<10t yr^−1^) and hence not visible at this scale.

Manta and devil rays are a group of large elasmobranchs that have taken up a filter-feeding pelagic lifestyle, similar to baleen whales or whale sharks. They belong to the family of eagle rays (Myliobatidae), but form their own subfamily, the Mobulidae, which includes two *Manta* species and nine *Mobula* species. Mobulids are subject to fishing pressure from bycatch [1], [2], [3] and are targeted for their gill plates [4], [5], [3], which are marketed as a medicinal product in Asian communities [3], [6]. According to the Food and Agriculture Organization (FAO), a total of 20,707 metric tons (MT) of mobulid species (FAO categories “giant manta”, and “manta/devil rays, nei”, have been landed by four countries from 1998–2010– with an average of 1,593 MT per year (Fig 1; note that there is also an undifferentiated FAO category of ‘rays, stingrays, manta nei’, which was not included because the proportion of mobulids in this category could not be estimated). Reported landings were dominated by Indonesia (82%) and Liberia (18%) and generally increased throughout this time period with peak landings in 2009. As is the case for other elasmobranchs [7], it is highly likely that reported landings only represent a fraction of total fishing-related mortality. In addition to incomplete catch data, little is known about the spatial and temporal abundance trends, exploitation rates, and trade volume of the Mobulidae at the global scale.

Similar to other elasmobranchs, mobulids are vulnerable to exploitation because they have slow life histories: they mature late, have low fecundity (produce one to two pups, have extended gestation period), and are presumed to be long-lived (up to 40 years for *Manta* spp.; [8], [9]). Furthermore, their ability to withstand fishing mortality is likely reduced because, at least for the species for which data exist (*Manta* spp.), many have small, fragmented and isolated populations [6], [10], [11] some of which appear to have declined [4], [5], [12]. Mobulids also often occur in surface coastal waters, making populations relatively easy to locate and exploit.

In recognizing conservation concerns for mobulids, some regions have introduced preemptive regulations to protect these species. All rays are protected from exploitation in the Republic of the Maldives, *Mobula* spp. and *Manta* spp. are protected in Mexico and Ecuador, *Manta* spp. are protected in Hawaii, and *M. birostris* and *M. japancia* are protected in New Zealand [12], [8]. In March 2013, both *Manta* spp. were listed on Appendix II of the Convention of the International Trade in Endangered Species (CITES). Despite these efforts, there are currently no international management plans in place to ensure the future of mobulid populations. However, because mobulids are mobile and do not remain within protected waters [11], international cooperation for their conservation may be necessary.

In addition to being fished mobulids have become profitable assets for the diving tourism industry in several parts of the world [13], [14], [15]. Mobulids are large (1–7 m disc width; [3]), charismatic animals that predictably aggregate in some shallow coastal waters, which makes them desirable and accessible to recreational divers. Because they are easily recognizable, aggregated divers’ observations provide a potentially valuable resource of data for coarse assessments of status and human use patterns of mobulid rays. Recreational divers’ observations have been previously used to describe local-scale patterns of manta ray distribution [16], as well as for other sharks and rays [17], [18], [19], [20]. Interviews of experienced recreational divers (i.e., instructors) summarizing past observations have also been aggregated to describe historical and contemporary patterns of shark and ray populations and human use where written-records are limited [21]. These types of data are especially valuable for data-poor areas or species, help to rapidly increase scientific knowledge, and motivate future research.

Here, we use a variety of data sources to describe and assess the contemporary distribution patterns, population abundance trends and threats of mobulid ray populations at the global scale. We present results from a directed global survey of divers’ observations to describe mobulid population trends and human use patterns. Divers are a valuable source of information for this purpose because of extensive spatial coverage, repeated sampling of the same dive sites over time, and the ease of identification of mobulids as a group [21]. We designed the survey to investigate global distribution, aggregation size and temporal population trends, as well as patterns of ecotourism, protection, fishing and markets of mobulids. To support these descriptions, and to provide more detailed observations, we used an existing diver database to describe regional distribution patterns at a finer spatial scale. Finally, we analysed life history characteristics of manta rays to determine their relative vulnerability to exploitation compared to other elasmobranch species.

## Methods

### Ethics Statement

Dalhousie University ethics committee deemed that the nature of the work (online survey of divers) did not require any approval or permits regarding human or animal ethics.

### Global Distribution and Human Use Patterns

Given that mobulids attract recreational divers [15], and the value of diver-derived data for describing broad trends in coastal biodiversity [21], we developed a focused online survey to gather information on the spatial and temporal trends and human use patterns of mobulids worldwide. The ‘eManta Survey’ was designed to collect the observations of experienced divers at a 10-degree latitude by 10-degree longitude cell resolution on a global scale (at the equator 10 degrees is 1110.56 km; Fig S1). The survey was distributed through available social-media outlets and diver organizations, and by contacting dive shops directly, focusing on the 173 spatial cells where manta rays are predicted to occur according to published species distributions on Aqua Maps [22]. We sent personal emails and phone calls to divers and dive shops in every cell possible, as identified from a Google search using land or sea features labeled on Google Earth within the cell (i.e. “Location”, “Dive Center” or “Diving”). We aimed for 5+ contacts per cell; however, 40 cells had fewer than 5 available contacts and 55 cells had no contacts listed or no mapped features that could be identified. Therefore, we sent survey requests to 125 cells, 85 of which had at least 5 contacts. Live-aboard dive boats and dive clubs were also included.

For each survey, divers were asked to select the cell, from a global map displaying all 648 possible cells, where they have logged a minimum of 200 dives and to summarize their dive effort and observations for that cell. As such, the survey was restricted to experienced divers who have typically logged thousands of dives in a particular area, and are familiar with local wildlife. Table 1 contains the list of questions asked that were relevant to this study. Data were summarized by cell and mapped at a 10×10 degree resolution. For each cell we calculated the number of records (i.e., participants) and dives (i.e., sum of all dives across all participants), median presence and absence of mobulids, average maximum school size excluding zeros, and median observed change in maximum school size (increase, decrease or no change) across all records. As for human uses, we mapped the median reported spatial extent of ecotourism, protective measures, fisheries that were observed to catch mobulids, and their occurrence in local markets.

**Table 1:** eManta survey questions.

Survey questions
1. From the map above, select the cell where you have the most experience. The questions that follow only refer to this cell. If possible, please fill out another survey for additional cells.
2. In this cell, how many dives have you done in total (approximately)?
3. In this cell, what were the first and last years that you were diving regularly? If you are still diving in this cell, select 2012.
4. Separating manta from mobula rays and identifying them to the species level is difficult. However, some circumstances allow for detailed observation. If possible, what species have you observed in this cell? Please use the identification figures below to be sure the same name is being used globally.
5. In this cell, what is the maximum number of individual manta or mobula rays you ever observed on one dive? Please choose your best and most conservative estimate (e.g. if 40–70 individuals, choose 40).
6. Have you personally observed a change in the maximum school size of manta or mobula rays in this cell?
7. In this cell, are you aware of any directed dives (ecotourism) for manta or mobula rays?
8. In this cell, are you aware of any restrictions on catching or fishing manta or mobula rays?
9. In this cell, have you personally observed anyone fishing or catching manta or mobula rays?
10. In this cell, have you personally observed any manta or mobula ray, or ray parts (e.g. wings, gill rakers, skins, etc), being sold, traded, or marketed? For example, these may have been individuals lined up on beaches, on ice, dried products in medicinal or curios stores, sold as purses or wallets, etc. *See photo below for manta ray gill rakers

### Fine-scale Distribution and Sighting Frequency

To describe finer-scale regional patterns in the distribution and sighting frequency of mobulids, we used dive-specific underwater visual censuses from three areas: Hawaii (HAW –12,411 dives), tropical eastern Pacific (TEP –4,222 dives) and tropical western Atlantic (TWA –118,548 dives; Table 2). Trained volunteer divers using the roving diver census technique (RDT) between 1993 and 2012 collected these data for the Reef Environmental Education Foundation (REEF, www.reef.org).

**Table 2:** REEF diver observation data.

Abundance indices	Hawaii	Tropical eastern Pacific	Tropical western Atlantic
Number of dives	12411	4222	118548
***Manta*** ** spp.**			
Total sightings	287	87	382
Total abundance (1,2–10,11+)	202,73,12	43,42,2	342,40
Mean sighting frequency (%)	2.3	2.1	0.3
Number of sites	538	488	7213
Number of sites present	73 (13.6%)	28 (5.7%)	135 (1.9%)
Number of sites with >1	27 (0.05%)	12 (2.5%)	24 (0.3%)
Number of sites with >2	5 (0.01%)	1 (0.2%)	0
***M. thurstoni***			
Total sightings	NA	20	NA
Total abundance (1,2–10,11+)	NA	5,9,6	NA
Mean sighting frequency (%)	NA	0.5	NA
Number of sites	NA	489	NA
Number of sites present	NA	13 (2.7%)	NA
Number of sites with >1	NA	12 (2.5%)	NA
Number of sites with >2	NA	4 (0.8%)	NA
***M. tarapacana***			
Total sightings	NA	9	4
Total abundance (1,2–10,11+)	NA	8,1,0	4,0,0
Mean sighting frequency (%)	NA	0.2	0.0034
Number of sites	NA	489	7214
Number of sites present	NA	7 (1.4%)	1 (0.01%)
Number of sites with >1	NA	1 (0.2%)	0
Number of sites with >2	NA	0	0
***M. hypostoma***			
Total sightings	NA	NA	25
Total abundance (1,2–10,11+)	NA	NA	22,1,2
Mean sighting frequency (%)	NA	NA	0.02
Number of sites	NA	NA	7214
Number of sites present	NA	NA	10 (0.14%)
Number of sites with >1	NA	NA	3 (0.04%)
Number of sites with >2	NA	NA	2 (0.02%)

Data are summarized for Hawaii, tropical eastern Pacific and tropical western Atlantic showing effort and mobulid sighting summaries.

For the purpose of this study we included all dives reported in the three focal areas. In the REEF database mobulid abundance was recorded as a categorical variable that corresponded to order-of-magnitude changes in fish abundance (e.g. 1 = 1, 2 = 2–10, 3 = 11–100 individuals sighted per dive). We report sighting frequency as the percent of dives where mobulids were present at a particular site. Where possible, temporal trends in mobulid sighting frequency (all species combined) were evaluated by generalized linear mixed models (GLMM, run with the glmmPQL package in R, www.r-project.org). Models included a binomial error structure (Bernoulli trails), logit link, year as a fixed effect, and site as a random effect (which assumes a different regression intercept but same slope for each site) to examine the overall trend while accounting for differences between repeatedly sampled sites. Only sites with mobulids present and a minimum effort of 10 dives per year were included in this temporal trend analysis (HAW and TEP>130 dives per site, TWA >200 dives per site). The REEF data collection began before the genus *Manta* was separated into two species (*M. birostris* and *M. alfredi*, [23]), and therefore we only report *Manta* spp.

### Species Vulnerability

Demographic analyses of species’ life history attributes can be used to quantify their relative vulnerability to added mortality (e.g., from fishing). Following methods described in detail by Smith et al. [24] and Ward-Paige et al. [25], [18], we used published biological characteristics of both *Manta* species to assess their vulnerability to exploitation relative to other elasmobranch species. Life history data were obtained from a number of peer-reviewed sources (Table 3) and included natural mortality (*M*), longevity (*w*), age at maturity (*α*), and fecundity (*b*, i.e. female pups per female per year, which was calculated from data on number of pups, gestation period and reproductive frequency, assuming a 50% chance of female pups). Natural mortality (*M*) was estimated from longevity using Hoenig's (1983) [26] formula:
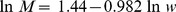



**Table 3:** Life history characteristics for *Manta* spp. in comparison with other elasmobranchs.

Species	Fmin	Fmax	Fper	Fave	Tmax	Tmat	r
Bonnethead shark	3.5	15.5	1	4.8	9.5	2.5	0.121
Blacknose shark	3	6	1.5	1.5	5.25	2.5	0.104
Atlantic sharpnose shark	1	9.5	1	2.6	10	3.35	0.098
Sandtiger shark	2	2	2	0.5	13.75	6	0.061
Grey nurse shark	2	2	2	0.5	25	6	0.058
Blacktip shark	1	10.5	2	1.4	15	7	0.054
Reef manta ray	1	2	2.5	0.3	32	6.75	0.05
Tiger shark	10	82	2	11.5	39	7	0.046
Spinner shark	4.5	15	2	2.4	12	7.5	0.045
Silky shark	6	13	1.5	3.2	25	8.5	0.045
Oceanic manta ray	1	2	2.5	0.3	30	9	0.042
Lemon shark	4	17	2	2.6	26	9.6	0.041
White shark	2	10	2	1.5	30	10	0.039
Bull shark	3.5	12.5	2	2	29.5	13.75	0.03
Scalloped hammerhead shark	21.5	35.5	1	14.3	26	15	0.027
Sandbar shark	3	13	2	2	31	16	0.026
Nurse shark	21	28	2	6.1	25	15.5	0.025
Basking shark	6	6	3	1	50	16	0.025
Smalltooth sawfish	15	20	1	8.8	70	17	0.022
Whale shark	16	300	2.5	32	70	27.4	0.016

Shown are attributes that relate to fecundity (F_min_ = minimum fecundity, F_max_ = maximum fecundity, F_per_ = fecundity period, F_ave_ = average fecundity), and life span (T_max_ = maximum age, T_mat_ = maturity age,). Intrinsic growth rates (*r*), are calculated from these data. Manta life history characteristics are reported in the methods section, and others are from references [18] and [24].

Survival to age at maturity (*l*_α_) was calculated from a variant of the Euler-Lotka equation:
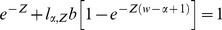
where *l_α,Z_* is survival to age at maturity when total mortality is equal to *Z.* Total mortality (*Z*) is set at twice the natural mortality (this condition is applied to minimize the effects of density dependence) and population growth is stable (*r* = 0) [24]. The intrinsic rate of population increase (*r*), which captures the population growth rate in the absence of density-dependent forces [24], was calculated as the value that satisfied the following variant of the Euler-Lotka equation:



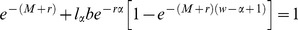



For *M. birostris* we used age at maturity (α) = 9 [9], longevity (*w*) = 30 [9], fecundity (*b*) = 0.3 (1–2 pups every 2.5 years; [8]) and for *M*. *alfredi* we used age at maturity (α) = 6.8 [12], [8], longevity (*w*) = 32 [12], [8], [27], fecundity (*b*) = 0.3 (1–2 pups every 2.5 years; [12]) (note that averages for each life history characteristic were used when multiple values were found). We were unable to find the required life history data published for any *Mobula* species.

## Results

### Global Distribution Patterns

**Figure 2:**
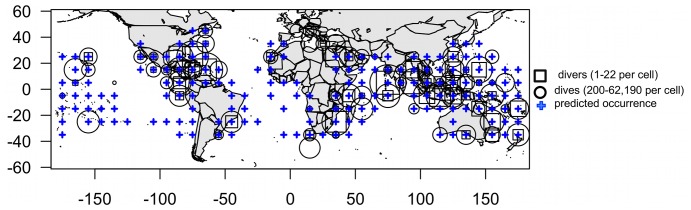
eManta diver survey effort with predicted occurrence of manta rays. The number of participants (divers), and the total number of dives per cell across all participants are indicated by square (1–22 divers per cell) and circle size (200-62,190 dives per cell). *Manta* spp. occurrence as predicted from published habitat suitability models [22], is indicated by crosses. eManta survey consists of 90 cells from 373 participants and 616,498 dives.

Between June and August 2012, 373 divers participated in our eManta survey, submitting data from 616,498 dives over 90 cells in all major ocean areas where mantas are expected to occur (Fig 2; [27]). Each of the 90 cells received data from 1–22 participants (mean = 4.1) and the number of dives per cell summed across all participants ranged from 200 to 62,190 (mean = 6,926) (Fig 2). Most divers (72%) started diving in their respective cell in the 2000’s, 25% in the 1980s–90s, and a few observations (3%) go back as far as the 1970’s. Assuming that no double counting occurred, a minimum of 5,802 individual mobulid rays was observed in this time period. Thirty-six percent of respondents reported only *Manta* spp., 15% reported only *Mobula* spp., 33% reported both *Manta* spp. and *Mobula* spp., and only 4% said they were unsure of the type of mobulid observed in their cell. The majority of submitted records to the eManta survey were located in eastern Australia, the Maldives, Thailand, Indonesia, Malaysia, the Gulf of Mexico, the Bahamas and the Red Sea (Fig 2).

**Figure 3:**
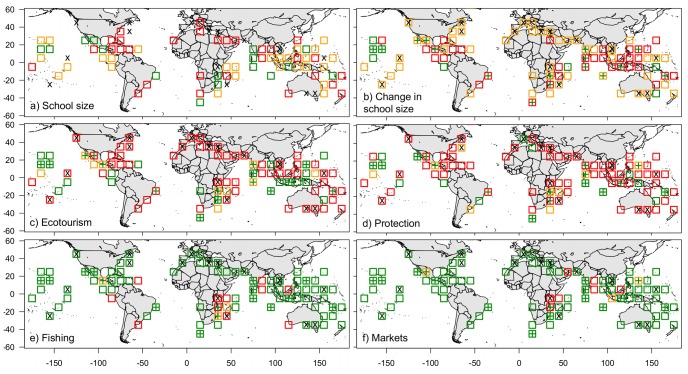
Global distribution and human use patterns from the eManta survey. In general green indicates positive (large school sizes, increasing populations, ecotourism and no fishing or markets), red indicates negative (small school sizes, decreasing population sizes, no ecotourism and markets); a) school size (x = zero, red = 1–5, orange = 6–20, green = >20 individuals), b) reported change over time in school size (green = increase, red = decrease, orange = no change), c) ecotourism (green = present, red = absent, orange = combination where not all respondents reported the same observation, d) protective measures (green = present, red = absent, orange = combination), e) fishing (green = absent, red = present, orange = combination), f) markets (green = absent, red = present, orange = combination). Green crosses in b–f indicate cells with large school sizes (>20 individuals from 3a), black crosses denote cells where mobulids were not observed. Note that only some part of each cell may be observed.

In the eManta survey, mobulids were reported in the majority (81%) of the 90 cells where data were submitted. The average maximum mobulid abundance (largest reported school size) for each cell ranged from 0 to 525 rays (mean = 18; Fig 3a), with the highest maximum school sizes in three cells occurring off the west coast of Mexico (525, 300, 63 individuals in three separate cells, respectively), Hawaii (42 individuals), and the Maldives (49 individuals). Of the cells with mobulids, 42% were reported to have no change in maximum school size over time, while 47% of cells were reported to have decreased and 11% reported increased school sizes (Fig 3b). Respondents reported ecotourism in 45% of cells (Fig 3c) and protective measures for mobulids in 32% of cells (Fig 3d). Personal observations of mobulids being fished were reported from 16% of cells (Fig 3e) and mobulid parts being traded or marketed were observed in 12% of cells (Fig 3f). While ecotourism and protective measures were scattered across the globe, fishing and marketing on mobulids appeared concentrated in the Indian Ocean (Fig. 3e,f). These areas were often adjacent to cells that had relatively high average school sizes (Fig 3a), and negative trends in school size over time (Fig. 3b). See tables S1–S4 for detailed comments and observations on the ecotourism, protection, fishing and market questions.

### Fine-scale Distribution and Sighting Frequency

**Figure 4:**
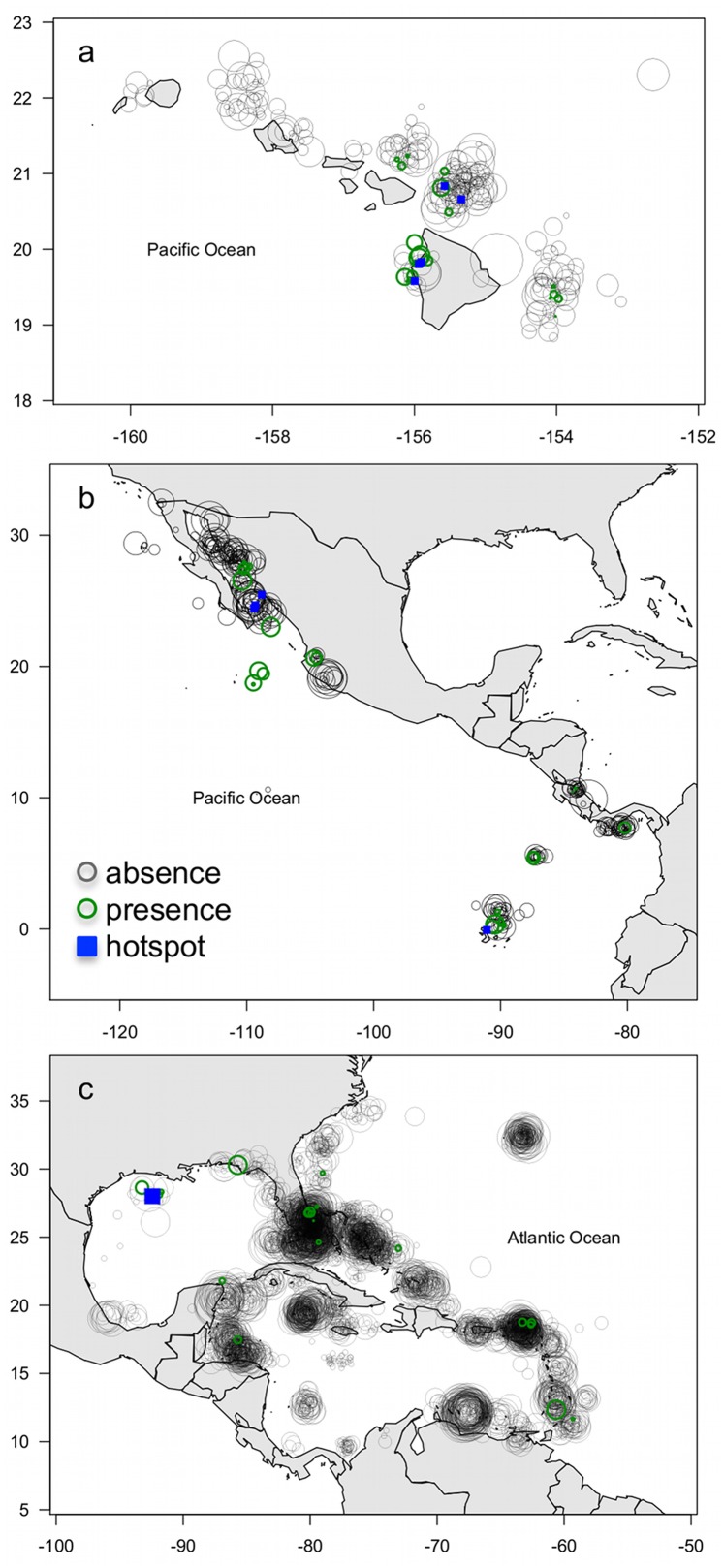
Fine-scale distribution and sighting frequency (SF) from REEF diver surveys. Circle sizes are proportional to effort (number of dives). Grey circles represent sites where mobulids were never reported, green circles represent sites where mobulids were present at any time and blue squares show sites where >10 individuals were observed at any point during the survey period. In (a) Hawaii SF = <1–99%, effort = 1–949 dives; b) tropical eastern Pacific: SF = <1–86%, effort = 1–152 dives, and c) tropical western Atlantic: SF <1–24%, effort = 1–2,039 dives.

Between 1993 and 2012, divers submitted 135,181 dives to the REEF database, covering Hawaii, the tropical eastern Pacific and the tropical western Atlantic (Table 2, Fig 4). Four mobulid species were reported including *Manta* spp. (likely a mix of *M. birostris* and *M. alfredi*), *Mobula thurstoni*, *M. hypostoma* and *M. tarapacana*. At least one of these species was observed on 811 dives (0.6%), with 188 dives reporting more than one specimen. Of the 7,996 sites visited, 257 sites (3%) had at least one sighting and 76 sites (1%) had more than one. Only three sites had more than one species of mobulid sighted.

Sighting frequency was highest in Hawaii where 73 of the 538 sites (14%) visited had at least one *Manta* spp. individual present between 2000 and 2012 (Fig. 4a). No *Mobula* species were observed in Hawaii. While controlling for effort (number of dives) and site, our GLMM analysis of 4,001 observations (dives) across 13 sites indicated that the probability of sighting a *Manta* spp. around Hawaii increased by approximately 10% per year during the last decade (0.095±0.04 SE, on the logit scale, p = 0.01). In the tropical eastern Pacific, three mobulid species were reported between 2000 and 2012, including *Manta* spp., *Mobula thurstoni*, and *M. tarapacana*. At least one of the three species was observed on 46 of 489 sites (9%), and 24 sites had more than one individual present (Fig. 4b). Only one site met our requirement of having mobulids present with more than 130 dives, but a year estimate (trend) could not be obtained due to the sighting only occurring in one year (2002). However, when relaxing the effort criteria and calculating trends for the top three dive sites, this analysis showed no significant change during the last decade (−0.318±0.17 SE, on the logit scale, p = 0.06) – note however the marginal significance level. In the tropical western Atlantic, the area with the lowest sighting frequency, three mobulid species were reported between 1993 and 2012, *Manta* spp., *M. tarapacana* and *M. hypostoma*. Mobulids were reported on 141 of 7,213 sites (2%), with 25 sites (<0.01%) having more than one individual. Our GLMM analyses of 16,579 dives across 30 sites showed no significant change over the last two decades (0.024±0.02 SE, on the logit scale, p = 0.25).

### Species Vulnerability

Based on an analysis of published life history characteristics we estimated the intrinsic rate of population increase for *M. birostris* as *r* = 0.042 and for *M. alfredi* as *r* = 0.050. Under density independent conditions, where populations are not limited by carrying capacity, populations are projected to decline when the instantaneous fishing mortality exceeds these values. Accordingly, *M. birostris* is slightly more vulnerable than *M. alfredi.* These *r* values are about average when compared to other elasmobranchs for which such data exist, and similar to tiger, spinner, or silky shark (Table 3).

## Discussion

By aggregating the observations of expert divers around the world, our eManta survey allowed us to rapidly broaden our understanding of global mobulid populations and human use patterns. Although there are limitations associated with this type of data (e.g., incomplete observations, large areas covered by cells), a comparison with the more detailed observations made by trained divers in the REEF survey, provides some confidence in our results, and improves our general understanding of spatial patterns and temporal trends of mobulids. This study demonstrates the rarity of aggregation sites for mobulids on a global scale and suggests that many populations appear to be declining. These observations, combined with increased landings of mobulids over the last decade (as reported to FAO), and low population resilience to fishing mortality, suggest that mobulid rays are in need of improved monitoring and protection measures.

A general concern with biological data collected by citizens is the accuracy of species identification. Since the REEF participants were individually trained in fish identification and most eManta participants reported being able to distinguish manta from mobula rays, we would expect identification to the genus level to be fairly precise. However, we chose to err on the side of caution and treat all mobulid species together because of similar appearances between species. Although this coarse level of grouping prevents analysis at the population or species level, this aggregated information may still be valuable for decision-making in more comprehensive applications that consider multiple species, and especially look-alike species (e.g. CITES).

One challenge of the eManta diver survey was to obtain negative observations – that is, garnering participation by divers that have never observed a mobulid. These observations are important for describing distribution patterns and temporal trends. Thus, because of a tendency for participation by divers that have actually observed mobulids, our survey could be biased towards overstating the presence and sighting frequency of mobulids. Similarly, areas where mobulids are rare or have recently declined may be underrepresented, if observations there are less frequently reported to eManta. It is also possible that mobulids may evade a diver, causing an underestimate of their presence. However, the occurrence of large numbers of mobulids in close proximity to hundreds of divers [14], [28], [16], and the fact that a number of scientific studies have used divers observations to investigate mobulid populations [6], [16], [10], suggest that such behavioral bias is likely not general.

Even with the large number of sampling events (>616,000 dives) mobulids were observed in only a small number of the world’s ocean cells (73/516), and where they were present, sighting frequency was generally very low (<0.01 mobulids per dive, on average). These patterns were corroborated by the REEF data, which also showed that even in areas where mobulids are frequently observed overall, they are only present on a few sites and that it is very rare for them to aggregate in large numbers or for multiple species to be observed in the same area. Spatial patterns and temporal trends reported to the eManta survey were complementary to the REEF data and showed comparable patterns and trends for the three regions where we had overlapping data. Hawaii and northwestern Mexico had relatively high sighting frequency with multiple aggregation sites, whereas the tropical western Atlantic was characterized by very low sighting frequency and a single aggregation site identified in the REEF data. According to both datasets, mobulid school sizes increased in Hawaii and were unchanged where they aggregate in northwestern Mexico. Unfortunately, the only aggregation site in the tropical western Atlantic in the REEF dataset was not covered by the eManta survey, however, the consensus among respondents was that school sizes were unchanged or decreased in this area. At the global scale, according to the eManta survey data, the majority of observed mobulid schools have remained similar in size or decreased over time. Negative trends were more visible at mobulid aggregation sites (>20 individuals per dive), where nine out of sixteen (56%) cells showed decreasing school sizes over time. Although no previous studies explicitly modeled changes in mobulid population abundance, our results corroborate reports that suggest populations may be in a state of decline [12], [8], [9], [29].

Approximately half the reported cells had mobulid ecotourism activity recorded (26% of all cells that are predicted to have manta rays), which were easily confirmed by a quick internet search for the terms “location” and “manta ray dive”. Divers described mobulid-directed ecotourism as “seasonal” and occurring at feeding and cleaning stations (see comments in Table S1), which is analogous to behaviours described in the literature [30], [16]. Manta-directed tourism can be economically important as respondents commented that “Sharks are the biggest draw and rays are a close second” and “people ask to see sharks and mantas when booked”. In addition, many divers reported cooperating with research organizations. These comments are supported by the scientific literature, whereas mobulids are reportedly worth US $73 million annually [13] with US $8.1 million annually to the dive tourism industry in the Maldives alone [14]. As well, recreational divers encounters have been used to describe mobulid populations in the Maldives [6] and Australia [16].

The degree of mobulid protection was resolved with some uncertainty. Respondents often commented on what they ‘believed’ to be true. This could be a result of a lack of awareness of existing regulations (fisheries regulations often do not apply to members of the diving community) or due to different opinions of what ‘protection’ entails. For example, divers from the Maldives stated that mobulids are protected because “Maldivian Law has banned the targeted fishing and export of manta rays”, while others stated that they are *not* protected because “It is prohibited under Maldivian law to export any ray products since 1996, but it is not actually illegal to fish for any ray species”. Despite this uncertainty, mobulids were reported to have some protection in about a third (n = 29) of the reported cells. And, since we have responses from all areas that have legal protection [12], the actual level of local protection is likely closer 17% of the 173 cells predicted to have manta rays. As an added caveat, the spatial scale of our 10°x10° cells is large, and the actual area under protection may only be a fraction of each cell.

Divers and fishers often have conflicting objectives, which can limit their interactions. Therefore, divers may not always be able to provide first-hand knowledge of fishing operations. However, many respondents commented on the prevalence of illegal fishing, the lack of enforcement where laws exist, and the need for legislation to protect mobulids from fishing activities in general. In some areas, mostly in the Indian Ocean and Indonesia, divers did report “dead carcasses on the beach brought in by fishermen” often “with their gill rakers removed”. They also reported on the numbers taken with “60 dead mobula in the water at any one time”. These observations are corroborated by the available literature. In the western Indian Ocean, for example, an estimated 53–112 t of mobulas and mantas have been taken annually as bycatch in the pelagic tuna purse-seine fishery [31]. In Mozambique, approximately 20–50 individuals were killed per annum in one 50 km stretch [32]. In Sri Lankan fisheries four mobulid species are regularly caught and marketed [33]. As well, in the Alor region of eastern Indonesia, where mobulids were historically harvested by harpoon from paddle or sail powered boats, fleet motorization has increased catches to 25–50 manta rays per boat and season, with and estimated total of about 1,500 manta rays per year [4].

Observations of mobulids being sold in markets were also limited. This may be due to some divers not attending markets because they “can’t bear to see it” or they are “only on a liveaboard”. However, they did report, for example, that mobulids are “sold everyday at the fish market”, are “for sale in Darajani Market, Zanzibar”, and that the “Lombok fish market, has several (10–20) mobulas and manta rays on the market every day”. Market observations included areas that are known to have large mobulid fisheries, such as western Mexico [34], Mozambique [32], Sri Lanka [33] and Indonesia [3], [4]. It is also expected that mobulids are not opportunistically sold in some locations where they are caught and are reserved for markets in Asia [12]. For example, mobulids were not observed being fished in the cell containing Singapore, southern Malaysia and north Sumatra, but they were observed in markets there, possibly indicating that they were fished elsewhere.

Life history characteristics indicate that manta rays would decline even at low levels of fishing mortality (*F* >0.05). Mobulid intrinsic rates of population increase (*r*), based on *Manta* spp. life history characteristics, are equal or are lower than many other elasmobranchs, such as the critically endangered grey nurse shark [35]. More generally, individual population sizes are estimated to be on the order of a few thousand individuals [6] and, in some cases ∼1,500 are taken per year [4] in just one location. As well, Dewar [4] found that the “general strategy of the Lamakera fishing fleet is to find a group of mantas and then return to the same group on subsequent days until they are gone”. These observations suggest that mortality rates in many areas likely exceed their intrinsic rates of increase. Likewise, local studies in Sri Lanka [33] and the Philippines [5] suggest populations are overfished and may be at risk of crashing.

Although many populations appear to be at risk of depletion, Hawaiian manta ray populations, which have been protected since 2009, present a more positive picture. In Hawaii, the REEF data showed increased sighting frequency where manta rays occur. Likewise, the eManta results show Hawaii to be the only region in the world with increasing mobulid abundance (two cells). Similarly, Deakos et al. [10] found survival rates of *M. alfredi* in Hawaii to be very high, with most individuals being resighted over a 5 year period. Combined, these observations suggest that Hawaiian manta ray populations are not being overfished and that legal protection can be effective for sustaining these populations (note that the isolated location of the Hawaiian island may also play a role).

The scientific literature, as well as our eManta reports of fishing and markets, highlight the paucity of reliable information on mobulid fishing mortality. Only four countries reported mobulid landings to the FAO since 1998. Indonesia landed the majority (82%), which is consistent with the literature [4], [12] and is supported by our eManta survey. However, other countries are not reporting their mobulid landings. For example, in Mozambique, Marshall et al. 2009 used mobulids caught in fisheries to study their reproductive ecology [23] and respondents to our eManta survey said they saw “Mantas being butchered on the beach in front of my dive centre, and mobula regularly being caught…”. Yet, Mozambique has not reported any such landings to the FAO. Likewise, Sri Lanka has never reported mobulid landings to the FAO, despite reports of fishing in our eManta survey and publications commenting on this fishery [9], [33]. As well, Dewar (2002) reported that an Indonesian village lands about 1,500 mobulids per year [4]. However, prior to 2002, only Liberia had reported mobulid landings to the FAO. Although it is possible that these mobulids are aggregated together with other species in the FAO “rays, stingrays, mantas nei” category, these records cannot be used to assess population trends and status of mobulid populations.

In summary, our study provides a broad overview of population trends and human use patterns of mobulids around the world, and supports previous studies in describing that mobulids are relatively rare within their range, are largely in a state of decline and are being fished in several areas that are not officially reported to the FAO. Despite some limitations of our data, and their application, these findings broadly support calls for increasing international conservation efforts for mobulids and helps to identify unprotected mobulid hotspots. Such regions could become a priority for increased monitoring and conservation initiatives. A paucity of data describing realized exploitation rates (percent of the population that is removed relative to the total population), suggests that this may be a priority for future research efforts. In the meantime, data collection needs to be improved with more accurate reporting of mobulid landings to the FAO – this may be as simple as providing species identification of catches. Finally, our study suggests that diver observations can be an important source of information in data-poor situations, and could be utilized more fully into the future.

## Supporting Information

Figure S1 **Map of cells used in eManta survey.**
(DOC)

Table S1 **Comments associated with the eManta survey question regarding directed dives or ecotourism in the cell (Question #7).**
(DOC)

Table S2 **Comments associated with the eManta survey question regarding restrictions on catching or fishing mobulids in the cell (Question #8).**
(DOC)

Table S3 **Comments associated with the eManta survey question regarding personal observations of anyone fishing or catching mobulids (Question #9).**
(DOC)

Table S4 **Comments associated with the eManta survey question regarding personal observations of mobulids being sold, traded, or marketed (Question #10).**
(DOC)

## References

[1] Storai T, Zinzula L, Repetto S, Zuffa M, Morgan A, et al (2011) Bycatch of large elasmobranchs in the traditional tuna traps (tonnare) of Sardinia from 1990 to 2009. Fish. Res. 109: 74–79.

[2] Amandè MJ, Ariz J, Chassot E, De Molina AD, Gaertner D, et al (2010) Bycatch of the European purse seine tuna fishery in the Atlantic Ocean for the 2003–2007 period. Aquat. Living Resour. 23: 353–362.

[3] White WT, Giles J, Potter IC (2006) Data on the bycatch fishery and reproductive biology of mobulid rays (*Myliobatiformes*) in Indonesia. Fish. Res. 82: 65–73.

[4] Dewar H (2002) Preliminary report: Manta harvest in Lamakera. Pfleger Institute of Environmental Research.

[5] Alava M, Dolumbalo ERZ, Yaptinchaya AAY, Torono RB (1997) In: Fowler SL, Reed TM and Dipper FA (eds). (2002) Elasmobranch biodiversity, conservation and management: Proceedings of the international seminar and workshop, Sabah, Malaysia, July 1997. IUCN SSC Shark Specialist Group. IUCN, Gland, Switzerland and Cambridge, UK. xv +258 pp. 132–147.

[6] Kitchen-Wheeler AM, Ari C, Edwards AJ (2012) Population estimates of Alfred mantas (*Manta alfredi*) in central Maldives atolls: North Male, Ari and Baa. Environ. Biol. Fishes. 93: 557–575.

[7] Worm B, Davis B, Kettemer L, Ward-Paige C, Chapman D, et al (2013) Global catches, exploitation rates, and rebuilding options for sharks. Mar. Policy 40: 194–204.

[8] Marshall A, Kashiwagi T, Bennett MB, Deakos MH, Stevens G, et al. (2011) *Manta alfred*i. In: IUCN 2012. IUCN Red List of Threatened Species. Version 2012.2. www.iucnredlist.org.

[9] Marshall A, Bennett M, Kodja G, Hinojosa-Alvarez S, Galvan-Magana F, et al. (2011) *Manta birostris* In: IUCN 2012. IUCN Red List of Threatened Species. Version 2012.2. www.iucnredlist.org.

[10] Deakos MH, Baker JD, Bejder L (2011) Characteristics of a manta ray Manta alfredi population off Maui, Hawaii and implications for management. Mar. Ecol. Prog. Ser. 420: 245–260.

[11] Graham RT, Witt MJ, Castellanos DW, Remolina F, Maxwell S, et al (2012) Satellite tracking of manta rays highlights challenges to their conservation. PLoS ONE 7: e36834. 22590622 10.1371/journal.pone.0036834 PMC3349638

[12] Couturier LIE, Marshall AD, Jaine FRA, Kashiwagi T, Pierce SJ, et al (2012) Biology, ecology and conservation of the Mobulidae. J. Fish Biol. 80: 1075–1119. 10.1111/j.1095-8649.2012.03264.x 22497374

[13] O’Malley MP, Lee-Brooks K, Medd HB (2013) The global economic impact of manta ray watching tourism. PLoS ONE 8: e65051. 23741450 10.1371/journal.pone.0065051 PMC3669133

[14] Anderson RC, Adam MS, Kitchen-Wheeler AM, Stevens G (2011) Extent and economic value of manta ray watching in Maldives. Tour in Mar Environ 7: 15–27.

[15] Anderson C, Waheed A (2001) The economics of shark and ray watching in the Maldives. Marine Research Centre; Shark News: 1–3.

[16] Couturier LIE, Jaine FRA, Townsend KA, Weeks SJ, Richardson AJ, et al (2011) Distribution, site affinity and regional movements of the manta ray, *Manta alfredi* (Krefft, 1868), along the east coast of Australia. Mar. Freshwat. Res. 62: 628–637.

[17] Bansemer CS, Bennett MB (2010) Retained fishing gear and associated injuries in the east Australian grey nurse sharks (*Carcharias taurus*): implications for population recovery. Mar. Freshwat. Res. 61: 97–103.

[18] Ward-Paige CA, Mora C, Lotze HK, Pattengill-Semmens C, McClenachan L, et al (2010) Large-scale absence of sharks on reefs in the greater-Caribbean: A footprint of human pressures. PLoS ONE 5: e11968. 20700530 10.1371/journal.pone.0011968 PMC2916824

[19] Ward-Paige CA, Pattengill-Semmens C, Myers RA, Lotze HK (2011) Spatial and temporal trends in yellow stingray abundance: evidence from diver surveys. Environ. Biol. Fish. 90: 263–276.

[20] Arzoumanian Z, Holmberg J, Norman B (2005) An astronomical pattern-matching algorithm for computer-aided identification of whale sharks *Rhincodon typus*. J. App. Ecol. 42: 999–1011.

[21] Ward-Paige CA, Lotze HK (2011) Assessing the value of recreational divers for censusing elasmobranchs. PLoS ONE 6: e25609. 22016771 10.1371/journal.pone.0025609 PMC3189927

[22] Kaschner K, Rius-Barile J, Kesner-Reyes K, Garilao C, Kullander SO, et al. (2010) AquaMaps: Predicted range maps for aquatic species. AquaMaps. Available: www.aquamaps.org.

[23] Marshall AD, Compagno LJ, Bennett MB (2009) Redescription of the genus Manta with resurrection of *Manta alfredi* (Krefft, 1868) (Chondrichthyes; Myliobatoidei; Mobulidae). Zootaxa 2301: 1–28.

[24] Smith SE, Au DW, Show C (1998) Intrinsic rebound potentials of 26 species of Pacific sharks. Mar. Freshwat. Res. 49: 663–678.

[25] Ward-Paige CA, Keith DM, Worm B, Lotze HK (2012) Recovery potential and conservation options for elasmobranchs. J. Fish Biol. 80: 1844–1869. 10.1111/j.1095-8649.2012.03246.x 22497409

[26] Hoenig J (1983) Empirical use of longevity data to estimate mortality rates. Fish. Bull. 81: 898–903.

[27] Kashiwagi T, Marshall AD, Bennett M, Ovenden J (2011) Habitat segregation and mosaic sympatry of the two species of manta ray in the Indian and Pacific Oceans: *Manta alfredi* and *M. birostris* . Marine Biodiversity Records 4: e53.

[28] MacCarthy M, O’Neill M, Williams P (2006) Customer satisfaction and scuba-diving: Some insights from the deep. The Service Industries Journal 26: 537–555.

[29] Notarbartolo Di Sciara G, Serena F, Mancusi C (2006) *Mobula mobular* In: IUCN 2012. IUCN Red List of Threatened Species. Version 2012.2. www.iucnredlist.org.

[30] Wilson S, Pauly T, Meekan M (2002) Distribution of zooplankton inferred from hydroacoustic backscatter data in coastal waters off Ningaloo Reef, Western Australia. Mar. Freshwat. Res. 53: 1005–1015.

[31] Romanov EV (2002) Bycatch in the tuna purse-seine fisheries of the western Indian Ocean. Fish. Bull. 100: 90–105.

[32] Marshall AD, Dudgeon CL, Bennett MB (2011) Size and structure of a photographically identified population of manta rays *Manta alfredi* in southern Mozambique. Mar. Biol. 158: 1111–1124.

[33] Fernando D, Stevens G (2011) A study of Sri Lanka’s manta and mobula ray fishery. Manta Trust 1–29. Available at www.mantatrust.org.

[34] Notarbartolo Di Sciara G (1987) Myliobatiform Rays Fished in the Southern Gulf of California (Baja California Sur, Mexico) (Chondrichthyes: Myliobatiformes). Mem V Simp Biol Mar Univ Auton Baja California Sur: 109–115.

[35] Pollard D, Smith A (2009) *Carcharias taurus* In: IUCN 2012. IUCN Red List of Threatened Species. Version 2012.2. www.iucnredlist.org.

[36] FAO Fisheries and Aquaculture Department, Statistics and Information Service FishStatJ: Universal software for fishery statistical time series. Copyright 2011.

