# Supplementary figures and images for: Global Population Trends and Human Use Patterns of Manta and Mobula Rays
Source: PLoS One. 2013 Sep 11;8(9):e74835. doi: 10.1371/journal.pone.0074835 (PMC3770565; doi:10.1371/journal.pone.0074835)

**Figure S1. Map of cells used in eManta survey.**

**
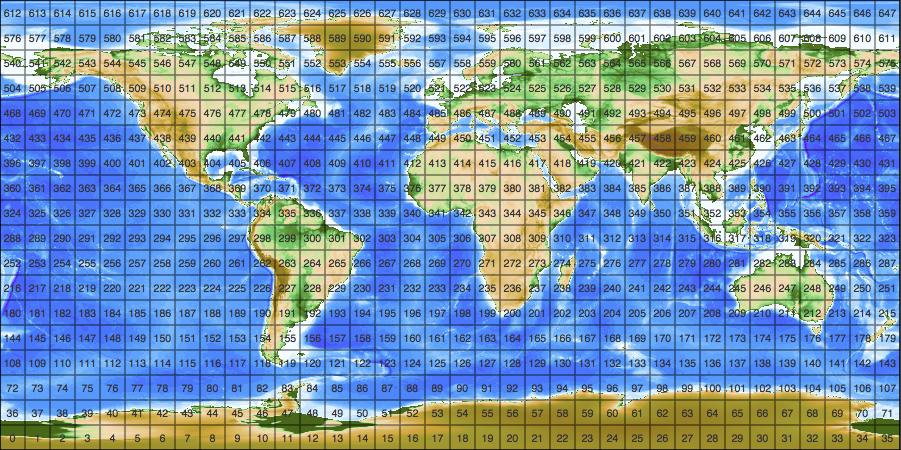
**

Supplement: Figure S1 — Map of cells used in eManta survey. (DOC) [file pone.0074835.s001.doc]
