# Supplementary material for: Global Population Trends and Human Use Patterns of Manta and Mobula Rays
Source: PLoS One. 2013 Sep 11;8(9):e74835. doi: 10.1371/journal.pone.0074835 (PMC3770565; doi:10.1371/journal.pone.0074835)
Supplement: Table S1 — Comments associated with the eManta survey question regarding directed dives or ecotourism in the cell (Question #7). (DOC) [file pone.0074835.s002.doc]

**Table S1. Comments associated with the eManta survey question regarding directed dives or ecotourism in the cell (Question #7).**

| Cell | Ecotourism |
| --- | --- |
| 372 | We have two sites where we go specifically to see rays, however mainly sting, then eagle, then manta rays...in that order of abundance |
| 369 | No, we live in Belize and Manta Rays are sighted very rarely, like i have only seen 3 in 15 years and over 1000 dives in this area. Never have i seen a Mobula Ray or even heard of other divers seeing them. |
| 229 | I published an article about diving with manta rays in Aug 89. I my knowledge it was the first article about it in a Brazilian magazine and since them the number of divers looking for a diving with mantas are increasing. The area was transformed in a State Marine Park mainly due the ocurrence of manta rays. |
| 249 | At lady Elliot islad |
| 249 | Im working as scuba diving instructor, and cooperate with the Project Manta of the UQ at Lady Elliot Island |
| 249 | Manta Lodge Stradbroke Island, Lady Elliot Island Resort both promote manta sightings as a highlight of their dives. |
| 249 | there are two main ones, sundive out of Byron Bay and North Stradbroke Manta Dive Centre |
| 279 | We regularly dive a cleaning station in the entrance of the Cocos (Keeling) Islands lagoon where we have seen up to 25 Manta alfredi |
| 333 | gaiding dide groups |
| 333 | Manta ray diving off catalina island in costa rica's shore & mobula ray sightings in cocos island , costa rica |
| 333 | people ask to see sharks and mantas when booked, so its part of the tour around Islas Tortuga, Puntarenas, Costa Rica |
| 333 | People go to Cocos Island to see large marine animals. Sharks are the biggest draw and rays are a close second |
| 333 | several dive shops do daily trips |
| 297 | Isla de la Plata (Plata Island) |
| 297 | There is a season every year when Mantarays become a big tourist attraction in the Machalilla National Park, in Ecuador. |
| 297 | we are conducting together with Dr Andrea Marshall a Manta ray research in this area the last 3 years? |
| 287 | Manta Reef (by mad Fish Divers and Matava Eco Resort) |
| 287 | Several areas of Fiji sell dive/snorkel trips just to see mantas. |
| 255 | Scuba dive in Kadavu, snorkel in Yasawas |
| 395 | Manta Ray cleaning station just off Boraa Bora, directed by Bora Dive Center |
| 361 | Manta Ray Experience , Kona Hawaii |
| 362 | Night manta Ray dive in Kona Hawaii. Also Day snorkels at a manta Ray Cleaning station where Mantas are seen 50%-60% of the time. |
| 397 | snorkeling and diving tours |
| 398 | I am going to assume this cell covers Hawaii. There are nightly dives for Mantas in the Big Island |
| 398 | Kona guided night dive, manta dive off Ukemehame |
| 398 | Manta Night Dive in Kona, HI; Guided boat dives out of Lahaina, HI. |
| 385 | dive with respect an don't touch explanation |
| 316 | don't understand the question |
| 316 | Manta Bay, Manta Point dive sites, Nusa Penida / Bali |
| 316 | Nusa Penida, Bali, Manta ray dives, feeding stations and cleaning stations |
| 316 | regular daily fun diving trips run by local dive centers |
| 316 | well known divesite Manta Point at Nusa Lembongan and Sunset point (or Manta Point) at the south of Gili Trawangan |
| 317 | Manta Awareness specialty dives. Survey dives for coral triangle and other organisations |
| 317 | Manta Point at Nusa Penida, Bali |
| 317 | Many dive centers in Bali promoted manta dive. |
| 317 | Nusa Penida which is an island about an hour to the east of Bali, Indonesia, is a cleaning station for Manta Rays. |
| 317 | Nusa Penida, Komodo |
| 317 | our dive center organizes trips to Manta Point Bali |
| 317 | Regular visits by liveaboards, including the ones I have run, to edutain guests on manta behavior patterns and appropriate diver behavior in the presence of mantas |
| 317 | Thé diving center we use in Bali ( atlantis international) is part of thé padi/ aware fundation and is affiliated to sea sheperd/ they are extremely careful when briging divers in diving spots where mantas are and do insist on bringing thé divers' attention to respecting marine Life (during pré and post dive briefing) |
| 317 | We have manta poit site at Nusa penida island |
| 317 | We regularly visit the manta cleaning stations |
| 317 | We run dives to Manta Point, Nusa Penida, Bali regularly, as do all dive centres on Bali. |
| 318 | Next to Bali, around Nusa Penida, there are two or more well known Manta Points, where one see Manta rays about 8 out of 10 times. |
| 318 | Not exactly ecotourism, but manta rays are a good arguments to attrack divers in this area |
| 318 | One sighting only. Tomkin Reef near Kampung Katupat Togean Islands |
| 318 | Raja Ampat is a dive paradise. There are specific sites that we went just to observe mantas |
| 318 | We, and many operators regularly take divers and snorkellers for viewing. |
| 319 | specific trip to cleaning and feeding area for Manta Alfredi |
| 319 | The area is known as Raja Ampat. It has several locations where Mantas congregate. |
| 319 | Travelled twice to sites called "manta sandy and manta slope" |
| 487 | There is no diving headed to see Mobula (no Manta in Mediterranean Sea, only Mobula mobular). But we are making tracked as part of our structure: "Corsica-Mediterranean Shark Research Group" (I am the president) and we follow up to Mobula around Corsica (486-487). See : http://corsica-requins-de-mediterranee.org/signalements/le-diable-de-mer-mediterraneen/. There is another association, we work with: "AILERONS" (it is mainly concerned with inland French). |
| 325 | I was working as a PADI Instructor on Bora Bora, French Polynesia. We took care that nobody was touching Manta Rays or Sharks or other animals. Every Diver had to do a check-dive on a sandy dive site before he was allowed to dive with us on the reefs. |
| 274 | research center and lots of cleaning stations |
| 352 | Two remote dive sites called Hin-Duang and Hin-muang where we used to go the see Mantas and Whale-sharks. |
| 353 | Pulau Selingan; an island off the NE coast of Indonesian Kalimantan. |
| 313 | All resort organize dives to "Manta points" |
| 348 | I am taking a guess that 348 is the Maldives. If it is not, then please correct the cell number. The liveaboard I was on were very strict (rightly so) when it came to Manta diving and had a number of protocols we had to follow. I would regard these protocols as ecotourism good practice. |
| 349 | A little from EPA Maldives www.epa.gov.mv |
| 349 | Anderson et al, 2011 |
| 349 | August - October are the season in Maldives to see Mantas feeding in the northern atolls (Baa) |
| 349 | Dive Trips to Manta cleaning and feeding stations. |
| 349 | Diving on known manta and mobula cleaning stations and feeding lagoons |
| 349 | During the Manta Ray season we run both targeted Snorkel and Dive trips to see Manta Rays |
| 349 | maldives are also popular for dive-boat trips for manta encounter. |
| 349 | Mostly at frequently visited cleaning stations, also at some feeding sites where large aggregations are observed |
| 349 | shallow reefs which are used as cleaning stations by the manta rays, and other bigger fish. |
| 349 | Specality courses |
| 349 | specific dive sites to cleaning stations |
| 349 | We did dives on divesite where we expect them in special times of the year |
| 349 | We regularly have trips specifically to "Manta Points" for either diving or snorkelling as the mantas are feeding or at cleaning stations. |
| 367 | Yes for Nari nari |
| 402 | sighting school of mobulas (underwater) as well as mobula breaching are common ,and gather customers for dive shops/snorkelling companies |
| 402 | You can see Mobula rays, but no trips are for diving with them, as it's unpredictable |
| 405 | I am sure there are though. |
| 405 | In Isla Holbox they have Whale Shark and Manta ecotourism - it is not "diving" but it is snorkeling. Mantas are one of the advertised species, although whale sharks are the primary species advertised. |
| 405 | Just snorkel. |
| 405 | There are two areas near where I live (In Cozumel, Mexico) that give tours to see mantas and whale sharks daily from the mid-June to the end of August, in Isla Mujere and Isla Holbox. We also see occasional Mantas here in Cozumel, but the majority of the time we see Southern Stingrays. In the Winter months we regularly see schools of Spotted Eagle Rays in Cozumel. |
| 368 | Catalina islands in Costa Rica is a cleanign station for manta rays |
| 356 | Yap is well known for Manta |
| 429 | In Palau & Yap |
| 237 | All dives in this area are guided dives. |
| 237 | Guinjata - Inhamabane Mozambique - Manta Reef |
| 237 | I have worked in dive tourism previously and now run a dive centre for the past 3 and a half years. Note, I have not been here continuously since 2003 but full time for the last 6 years |
| 237 | I specifically sell dives to guests to go and see both Manta's and Devil Rays |
| 237 | manta ray hot spot with lots of directed tourism for both species of Manta |
| 237 | on one of the reefs we have a few cleaning stations where mobulas come and we specifically bring divers there to see them. - same for other reefs where we have mantas cleaning stations |
| 237 | we organize these dives |
| 237 | yes. marine mega fauna research center works close with the dive centers |
| 237 | Zavora bay as well as Tofo offers dives with both Manta alfredi and M birostris all year around. There is extensive research done on the animals at both locations |
| 284 | Project Manta research work which takes on volunteers |
| 391 | Yap is a Manta Sanctuary |
| 392 | German Channel & Devilfish City are both tourist dives for reef manta rays |
| 355 | During my professioal engagement in Palau (as a dive guide and instructor) I personally conducted approximately 300 dives at the top mant spot, German Channel and about 25 dives in other sites. |
| 355 | During my professional engagement as a dive guide in Palau, I had the opportunity to guide about 300 dives in German Channel, Palau's prime spot for manta encounters. |
| 355 | We offer dives to German Channel regularly for our guests here at Sam's Tours |
| 354 | Divecenters in Donsol and Malapascua |
| 354 | Sites where Mantas are regularly seen but not directed exists in the Philippines. One site called Manta Bowl in the Burias Pass is directed for Mantas though |
| 354 | Tubbataha live aboard scheduled dives where I was the dive master |
| 390 | manta rays are part of the big attraction for the established dive tourism here in malapascua island, second to the pelagic thresher shark that visit regularly a submerged sea mount known as monad shoal. |
| 390 | There is a dive site called Cervera Shoal off Pamilacan Island, Bohol, Philippines. It has been possible to see Mantas/Mobula here before but it is very rare now. |
| 401 | I worked on the Nautilus Explorer live aboard, which markets this area heavily toward divers who want to interact with mantas. |
| 321 | The Devils Hwy. A narrow channel with strong current that rays feed in. Divers sit and watch rays fly by. |
| 163 | Dived on Manta cleaning stations with holidays divers.And the divers are briefed not to go on the cleaning station and not to touch the manta's at all. You can see over 20 to 30 mantas on some dives with an average of 10 to 15 on most dives. Schooling Mobulas have been seen in groups larger than 15 to 40 at a time during the seasons. |
| 201 | A few spots where try to see rays but not guaranteed |
| 211 | eagle and sting rays |
| 273 | In the Bazaruto Archipelago there is an area known as San Sebastian which has 3 sites with Manta cleaning stations. On 2 Mile Reef there is a dive site known as Devils Dance because of the Mobulus cleaning stations |
| 351 | 2 dive sites have cleaning stations for mantas. However no mantas were spotted this year at these 2 specific sites |
| 351 | Dive operators promote particular dive sites with sitings of mantas just diving hoping to see them. |
| 351 | Dive trips are specifically marketed to interact with mantas. |
| 351 | I m diving instructor |
| 351 | many dive operators market the Island Koh Bon as a manta cleaning station and is hughly popular spot to dive during the high season. Also Koh Racha Noi ( the south tip) is also marketed as a mata cleaning station during the high season |
| 351 | There are dive centres in Khao Lak who do Manta counts and submit pictures taken to Ecocean.org. Wicked Diving does special educational trips about Manta rays. |
| 387 | many liveaboards visit this site |
| 387 | Marginally less sightings |
| 387 | My dive centre, Blue Guru, runs trips with eco-education currently focused on sharks & turtles. However we plan to add eco-education for mantas and already report sightings to a facebook page monitoring manta rays in Andaman Sea. |
| 387 | Sea Dragon Dive Centre, Khao Lak, offer weekly day trips out to Koh Bon to see Manta Rays for divers and snorkellers. |
| 387 | Similan Island is visited by Manta rays in the fall of each year! |
| 387 | Similans Island are a clean station |
| 387 | There is no ecotourism specifically targeting rays. |
| 371 | Used to see many many in Speyside, Tobago and so we used to advertise to come see them and dive with them. |
| 371 | We go diving with them watching every full moon they come in to feedon the plankton |
| 245 | Several firms specialise in manta trips in ningaloo |
| 271 | Not companies but maldives. Bali, and Great Barrier Reef have dives for Mantas. |
