# Supplementary material for: Global Population Trends and Human Use Patterns of Manta and Mobula Rays
Source: PLoS One. 2013 Sep 11;8(9):e74835. doi: 10.1371/journal.pone.0074835 (PMC3770565; doi:10.1371/journal.pone.0074835)
Supplement: Table S2 — Comments associated with the eManta survey question regarding restrictions on catching or fishing mobulids in the cell (Question #8). (DOC) [file pone.0074835.s003.doc]

**Table S2. Comments associated with the eManta survey question regarding restrictions on catching or fishing mobulids in the cell (Question #8).**

| Cell | Protection |
| --- | --- |
| 369 | There is not a law in Belize but Belize is really into ecotourism and marine life protection, just by word of mouth. |
| 229 | At Laje de Santos Marine State Park is proibihed any fishing or catching. |
| 229 | It is a conservation area and fishing is forbbiden. |
| 229 | The manta rays are extinguishing. |
| 249 | Government regulates shark fisheries and there is no current manta fishery in australia. |
| 249 | I work in the green zone of Great barrier reef marine park. |
| 249 | It is part of Great Barrier Marine Park |
| 249 | Mantas are protected |
| 279 | Mantas are not sort for food on the Cocos (Keeling) islands |
| 333 | Cocos Island is both a Costa Rica National Park and a World Heritage Site. Laws are in place to protect it. These are not always enforced. |
| 297 | In august of 2010 a government decree (decree 093) was issued prohibiting any fishing of mantarays, mobula rays, or any kind of rays. |
| 297 | The Ecuador Government put a special law to protect Mantas and Mobulas |
| 287 | I WISH Fiji had Shark and Manta protection policies! |
| 287 | Not in Fiji as far as I know |
| 486 | They are protected under Spanish law since 2010. |
| 325 | Waters off of Palmyra Atoll are protected as a US National Wildlife Refuge (as well as a Marine National Monument). |
| 361 | Hawaii State Law |
| 362 | Some legislature was passed in (2010?) for Hawaiian Waters stating that it was a finable offence to Harrass or kill the Mantas ( Or something like that) |
| 398 | Hawaii put Giant Manta on the Protected List in 2009 (I believe). |
| 398 | Mantas are protected |
| 316 | The 3 Gilis are inside a MPA but law enforcement is poor. Local fisherman are willing to change but need to be compensated somehow. |
| 316 | We are aware that fishing is prohibited in many areas of our marine protected area, but even if we work closely with the government there is no control |
| 317 | I understand it is not allowed but it seems to be a gray area |
| 317 | In Komodo national park.It's protected but the area around here such as Lombok,Sumbawa,etc.I saw photos from friends or journalist that they catch the Manta and Mobula every single day now.So we concern about in the future if they come to Komodo National Park to catch the Manta especially on low season in the south of Komodo where its located Manta site name "Manta Alley" which is the famous Manta aggregation site in Komodo.On low season not so many diving boat come here and its far away from patrolling area for ranger.Sometime we saw fishing boat from the nearby island came here. |
| 317 | Sould be but fisherman from Lombok still catch them! |
| 317 | The area is a designated Marine Protection Area with designated fishing, diving, water sports and no-go zones |
| 317 | The Komodo National Park is mostly a no-take zone. However, I have heard of dead mantas being sold in the local fish market. This park is, frankly, very poorly managed. |
| 318 | Komodo is a National Park |
| 318 | Not on the full area. But some dive centers try to protect the small area where they work (conservation center, promotion of environment good practices) |
| 318 | within national park boundries they are supposed to be protected. enforcement is lacking. |
| 319 | 4 areas are marine protected areas but areas are to big and not enough patrol/manpower and lots of corruption. |
| 319 | Supposed to be a Marine Park |
| 451 | Mobula Rays have been protected in Malta since 1999. |
| 487 | Barcelona Convention, Annex 2 Bern Convention, Appendix 2 |
| 274 | well trying to get restrictions |
| 352 | There were marine protected areas where all fishing was supposed to be banned 2 nautical miles off any shore. Although this was not enforced with any real authority |
| 313 | Just not done, culturally or otherwise. The local feed mainly on tunas they do not consider Manta/Mobula as food or profitable catch |
| 313 | Protected in Maldives |
| 349 | as you know...at hanifaru bay, fishing is strictly forbidden. |
| 349 | Fishing not allowed, if caught will get fined |
| 349 | I am not 100% sure but I think the same regulation as for sharks fishing applies to Manta rays, it is prohibited to fish sharks in MAldives |
| 349 | I believe is forbidden to catch sharks or mantas in Maldives |
| 349 | It is prohibited under Maldivian law to export any ray products since 1996, but it is not actually illegal to fish for any ray species. |
| 349 | It's prohibited |
| 349 | Maldivian Law has banned the targeted fishing and export of manta rays |
| 349 | no fishing on sharks or big rays. (still happens because there is a market for it) |
| 349 | On the popular diving reefs any kind of fishing is restricted, but here illegally some fishing occured on sharks, but no manta fishing observed. |
| 402 | sportfishing is allowed in soutehern Baja (cabo san lucas) ,but commercial fishing is only legal 20 miles off the coast |
| 405 | Florida Fish and Wildlife Conservation Commission 68B-44.008 Prohibited Species; Prohibition of Harvest, Landing, and Sale. (1) No person shall harvest, possess, land, purchase, sell, or exchange any or any part of these species: (dd) Manta ray (species of the genus Manta and Mobula). |
| 405 | Florida Keys National Marine Sanctuary - it is illegal |
| 405 | Manta rays are a protected species in Florida waters. |
| 405 | protected |
| 368 | Part of the area is national park |
| 237 | At the moment it is not illegal to fish mantas or mobulas in MNozambique, but laws to protect are in process |
| 237 | However there is no inforcement. |
| 237 | I am aware that there are no restrictions |
| 237 | Marine protected area (Aliwal Shoal MPA) |
| 237 | marine protected area prohibits the capture of non-game fish species |
| 237 | NO restriction |
| 237 | There are no restrictions in place to protect manta rays in Mozambique |
| 237 | they trying hard to do a conservation area out of it |
| 237 | we have a area around Vamizi Island which is a no fishing zone not even catch or release. |
| 284 | Nil fishing in GBR |
| 215 | No restriction or protection of rays in New Zealand |
| 391 | Absolutely forbidden as a marine sanctuary |
| 392 | Illegal to fish for Manta in Palau. |
| 392 | There is a law in Guam against the possession, selling, offering for sale, trading, or distribution of shark fins, rays and ray parts. |
| 355 | Palauan law prohibits the catching of both sharks and mantas |
| 355 | Reduction in asia |
| 354 | BUNAKEN NATIONAL PARK FISHING NOT ALLOWED HOWEVER I HAVE NEVER SEEN MANTA ONLY EAGLE RAYS |
| 354 | Manta protected Mobula not |
| 354 | Protected reef patrolled by security |
| 354 | Protected under Philippine law, but usually no sanctions imposed |
| 390 | I heard that it is allowed to catch mobula rays, but they also catch mantas because it is difficult to tell the difference |
| 390 | Manta ray is illegal to catch, however mobula are not. People still catch manta and get away with catching it because of their similarity and claim they are the mobula to authorities. The Dept. of Environment & Natural Resources of the Philippines is one of the most corrupt depts. in the Philippine govt so it can be easy to get away with those sorts of things. |
| 390 | the provincial government of cebu has recently passed an ordinance declaring several marine megafauna as protected but implementation is a different thing. |
| 401 | The Revillagigedos archipelago is a World Heritage site and all fishing is illegal within 200 miles. |
| 417 | MPA |
| 417 | No fishing is allowed in all the marine parks. |
| 163 | I am not aware that it is by law but from my experience in traveling is different atoll the fishermen themselves do not bother about mantas or Mobulas. |
| 201 | Marine reserve - no fishing or catching allowed |
| 213 | Mantas protected along the Australian east coast |
| 213 | Not 100% sure but http://www.dpi.nsw.gov.au/__data/assets/pdf_file/0020/202349/NSW_SWG_2012.pdf |
| 273 | Mozambique has a law forbidding the taking of Manta Rays |
| 351 | It is suppose to be a marine park however this does not stop the fishing boats and nets are often seen on the dive sites. |
| 351 | Koh Bon is National park area so no fishing is allowed. But needless to say fishing does occur in these areas eespeecially during the low seeason when there are no dive operators allowed to dive here. I dont think there are any restrictions around Racha Noi as many sport fishing boats frequent these areas |
| 351 | Officially, the water around the Similan and Surin islands are protected in a 3 mile radius, but there is fishing going on within. Personally observed it many times. |
| 351 | Similan National Park |
| 387 | All fishing is restricted in the Similan and Surin national parks, of which Koh Bon and Koh Tachai are part, but we always saw illegal fishing vessels long-lining and dropping fish cages in the parks during the diving season from November - April. |
| 387 | National Park zone 3km around Surin Islands, Richelieu Rock, Koh Tachai, Koh Bon & Similan Islands. However fishing boats fish right on the perimeters between these islands. |
| 387 | There are no restrictions on catching manta or mobula rays. |
| 371 | In MPAs |
| 371 | We have no laws here regarding them, nor is the area we used to see them most protected despite numerous calls for same ! (Speyside, Tobago) |
| 192 | It is a dive park and no fishing should be allowed. |
| 406 | Protected in U.S. waters |
| 406 | socialy not accepted. Other fisheries more finacially productive. Limited sitings. Prabable government restrictions, Bahamas. |
| 286 | dive sites in our area are owned by communities who protect their sites in return for a custom fee (per diver, per dive) from tourists |
| 473 | there are restricted areas in the gulf islands of BC that are protected marine habitats ... it is illegal to fish in those waters. Certain species in BC waters are off limits all together. |
| 310 | In fact i am not sure as Kenya has not many restrictions in general apart from No Fishing Zone within the marine parks |
| 348 | This needs a "I don't know" answer for this. Please take this answer as "I have no idea, I don't know". |
