# Supplementary material for: Global Population Trends and Human Use Patterns of Manta and Mobula Rays
Source: PLoS One. 2013 Sep 11;8(9):e74835. doi: 10.1371/journal.pone.0074835 (PMC3770565; doi:10.1371/journal.pone.0074835)
Supplement: Table S3 — Comments associated with the eManta survey question regarding personal observations of anyone fishing or catching mobulids (Question #9). (DOC) [file pone.0074835.s004.doc]

**Table S3. Comments associated with the eManta survey question regarding personal observations of anyone fishing or catching mobulids (Question #9).**

| Cell | Fishing |
| --- | --- |
| 372 | very seldom but seen about 3 mantas killed on beach over last 20yrs |
| 229 | Accidental manta ray death due fishing net entrapments are rare but reported. |
| 229 | Manta rays sometimes are catch by nets. But, it is not intentionally. |
| 249 | Bycatch of manta alfredi from gold coast shark nets. |
| 333 | I have seen illegal fishing inside the boundaries of the park. These longlines catch rays as well as many other species. |
| 297 | In late 2009, early 2010 there was an explosion of fishing activities directed to mobula rays in many fishing ports in Ecuador. The target was for the meat that was sold to Peru according to the fishermen. |
| 297 | Isla de la Plata (Manabi province, Ecuador), Ayangue (Santa Elena province, Ecuador), Cope (Santa Elena province, Ecuador), Galapagos Islands (Galapagos province, Ecuador). |
| 297 | not since the Ecuadorian Government Protect them |
| 287 | But I have heard this happens frequently in Fiji. |
| 287 | http://www.fijitimes.com/story.aspx?id=209940 |
| 287 | In Suva you can find the local inshore fishermen selling them |
| 386 | Fishermen. It's India. |
| 316 | Fishermen catch manta rays for food and gills in Lombok area. we have paid local fishermen to release them when they were still alive. |
| 316 | I once observed a fisherman with a manta tied to the side of the boat. We paid the fisherman to release the manta as it was still alive and able to swim away. Head of the island discussed with him to not do it again. |
| 316 | Often rays have hooks in fins/frontal lobes |
| 317 | fishermen drag netting |
| 317 | In Lombok a neighboring island the locals are fishing them and selling their gills to China for Chinese medicine. |
| 317 | Not me personally but i know that's happening!!! |
| 317 | seen mantas on land after caught by fishermen on Bali (Amed) |
| 317 | Visited the Tanjuar Fishery an they are catching both around Sumbawa, Lombok and according to captains on Lembongan, also have been seen coming to Penida |
| 318 | From 1996 to 1998 as good as the whole manta polupation of North Sulawesi was fished out by tiger trap nets. Nets have been removed and we did not see big rays for many years. Now 15 years later we are under the impression we see big rays slowly but surely more often then after the trap net disaster. |
| 318 | I have seen them beeing finned on the beach. Horrible! |
| 319 | foreign (indonesian) fisherman that specifically come to hunt for sharks and manta/mobula species for the chinese/taiwanese market. |
| 309 | Locals, and we have fishing trawlers of the coast |
| 310 | 1 mobula ray at mnemba atoll, zanzibar a marine park none the less |
| 310 | Deep sea fishing from Big game fishing comanies and locals (they take what they get) |
| 310 | Local fishermen fish them. They are being sold for food at the fish market |
| 310 | Once about 7 years ago we have seen a local very small fishing boat with a Manta of about 2.5 meter |
| 353 | caught, just last week by a fisherman. |
| 353 | Mantas get caught in trawl nets. They turn in the fish markets all the time. |
| 313 | local maldivian fisherman are catching manta rays, cut thier top fins adn sell them as shark fins to asian market. |
| 368 | not them being pulled out of the water, but we have seen many attached to line, few we managed to safe |
| 405 | I am aware that some fishermen catch them off Isla Mujere and off the shores of the Yucatan Peninsula. |
| 237 | directed and incidental catches of mantas particularly Manta alfredi |
| 237 | I have seen local fishermen catch large numbers of devil and eagal rays |
| 237 | Local fishermen are not generally targeting mantas or mobulas, but will keep them if caught in nets. We observe catching of these animals every few months. |
| 237 | Locals, seems to be by catch but still sold. Manta meat gets approx 60 cents US a kilo. Netting is an increasing problem. |
| 237 | mobulas, mostlly juvenile, fished by 100s |
| 237 | the government gave the poor fishing people a new boat, since that they coming back with lots of sharks and mobula rays. |
| 237 | There are illegal fishing actavities in this area. Also the locals catch Mantas and other rays as by-catch which then get sold off localy in the market. |
| 237 | thousands of devil rays, few mantas |
| 215 | Stories of commerical fishermen chopping manta tails off roughly 20 years ago (nothing recently) |
| 354 | Mobulid rays are fished in the Philippines 1. For local consumption (fins and meat) 2. Traded (gills) |
| 390 | I once saw a small fishing boat return to shore with a catched manta ray |
| 390 | It is very common to see dozens of mobula (few manta) caught and bought to the Baclayon Port to be taken elsewhere for the drying process. It is possible to see maybe 60 dead mobula in the water at any one time which is very, very disturbing. |
| 401 | We caught an illegal fishing boat with shark fins on board - not 100% sure, but I am confident to say that they killed everything they caught on their long line, including mantas. |
| 417 | Once with a long line. |
| 417 | Russians&saudi's mostly.illegal fishing tours for well paying guest,unfortunatly |
| 417 | small boats, local fishermen, showed us a small manta (~70cm) that they'd caught in a net. They are also seen occasionally in the Jeddah fish market. |
| 419 | Fishing Rays is common. but we don't have manta rays. we have eagle rays and grey rays |
| 163 | I did find a Manta dead by getting it self caught on a rope.I cut it loose myself and let it sink in to the deep blue. |
| 350 | Clarification - Seen rays caught on fishing nets. This is NOT specifically fishing for rays. |
| 350 | they turn up at fish stalls. |
| 273 | I have seen the dead carcasses on the beach brought in by fishermen |
| 273 | In the local fish market we see rays being cut up making hard to tell which is which, but I suspect both. |
| 351 | Tarutao national park, thailand is supposed to be protected but this is not the case. Like in many other Asian countries corruption from fishing companies make the rangers of the national park close their eyes ... Giving all the freedom needed to fish any animal, including mantas, whale sharks. |
| 387 | But I know other instructors who have seen manta ray landed at Tap Lamu pier near Similan Islands. |
| 387 | I have not witnessed manta rays being fished but have regularly witnessed illegal fishing activity in the national parks around the Andaman Coast of Thailand - sharks are felt to be particularly vulnerable and I have seen photos of manta caught in fishing lines at Koh Bon. |
| 387 | Similan is full of Fishing boats. Don't know if they catch manta rays |
| 371 | Not directly fishing them, but has seen three instances where the wings have been cut and the rest of the body is been eaten by other fishes. |
| 371 | Riding and Harassing them yes, fishing and catching no ! |
| 192 | Fishing in general, not for manta or rays. |
| 209 | Has observed rays being caught by accident |
| 238 | I saw a boy one day holding up a small manta on the beach |
| 274 | locals chat manta and rays often |
