# Supplementary material for: Global Population Trends and Human Use Patterns of Manta and Mobula Rays
Source: PLoS One. 2013 Sep 11;8(9):e74835. doi: 10.1371/journal.pone.0074835 (PMC3770565; doi:10.1371/journal.pone.0074835)
Supplement: Table S4 — Comments associated with the eManta survey question regarding personal observations of mobulids being sold, traded, or marketed (Question #10). (DOC) [file pone.0074835.s005.doc]

**Table S4. Comments associated with the eManta survey question regarding personal observations of mobulids being sold, traded, or marketed (Question #10).**

| Cell | Markets |
| --- | --- |
| 237 | Mantas being butchered on the beach in front of my dive centre, mobula regularly being caught, here is more the sharp end, they sell else where. |
| 237 | Mostly mantas have been targeted for meat but there have been several individuals seen with their gill rakers removed so it is possible that they are selling them to the Chinese as there are many buyers for shark fins in the region |
| 237 | no gill, but mobulas lining up on the beach of piled up in wheel barrels - mostly sold on the local market, for meat |
| 237 | we are located right on the beach, where the fisherman come back with their catches of the day... |
| 249 | I have personally seen gill rakers in a chemist in Shanghai, China. |
| 273 | I have not seen evidence that fishers are specifically extracting gill rakers, but it could be the case. |
| 273 | Only offered on the beach for food |
| 284 | I have done most of my diving out on the Great Barrier Reef off Cairns and have unfortunately only ever seen one manta ray |
| 287 | In Suva you can find the local inshore fishermen selling them. Whole small rays and parts of not just "bits" |
| 297 | It happened during some months, maybe a year, where you could see dozens or even hundreds of mobula rays daily being landed. It stopped almost completely since decree 093 went into effect. |
| 309 | In the market in Stone town! |
| 310 | Manta rays have been seen for sale in Darajani Market, Zanzibar. |
| 310 | sold as fresh catch to the local market for consumption |
| 310 | sold everyday at the fish market |
| 313 | top fins are sold to asian market as dried shark fins |
| 316 | Fishing port in East Lombok, Indonesia |
| 316 | In Lombok we have one of the biggest fish market (tanjung Luar) for sharks and rays... everyday many are brought by boat and sold to chinese people.... |
| 316 | Lombok, fish market, has several (10-20) mobulas and manta rays on the market every day. |
| 316 | Markets in Lombok sell rays and sharks in huge numbers. |
| 317 | But I have not visited the local market |
| 317 | Fish Market in Bali!!! And in Lombok |
| 317 | not personally, but it has been observed by close friends. I don't go to the market anymore.... can't bear to see it. |
| 317 | Tanjuar Fishery visit as mentioned earlier |
| 318 | have seen manta and mobular ray in local fish markets. |
| 333 | products offered including meat |
| 349 | Haven´t been on male only on a livaboard. |
| 350 | 100s of 1000s of sting rays being dried |
| 351 | at phuket market with shark too |
| 351 | Fish market on the mainland sell mantas and sharks. Locals love to eat them. I ve even been offered dead black tip sharks at the dive centre by local fishermen... |
| 351 | Fish markets on Phuket serve a varieety of rays and sharks |
| 351 | I usually see it in the wet market. |
| 351 | In Thai and Indonesian markets. |
| 351 | mantas for sale at market in Ranong |
| 351 | phuket fishing pier |
| 351 | purses and wallets, but not sure if real or fake |
| 353 | Purses. |
| 353 | Sold for food in the fish markets. |
| 354 | Fishery exists mainly around the Bohol-Mindanao Sea |
| 354 | I AVE SEEN ONLY AT THE INTERNATIONAL AIRPORT SUKARNO HATTA IN JAKARTA A STORE THAT SELLS FINS FROM ALL ENDANGERED SPECIES AS WELL AS DRIED SEAHORSES! |
| 386 | Straight to fish market |
| 387 | Not personally but know instructors who have. Personally I have seen juvenile blue spotted rays on Kuraburi night market stalls every day. |
| 390 | In the Philippines they dry and eat the manta/mobula meat which looks much like beef jerky. It is a prize catch for fishermen as they can earn about US$45 / kg compared to other fish species which may only be US$3-4/kg. If you go to Pamilacan Isl. you can see the rays being dried on drying racks. It is hard to curtail on this island as the head official of the island is one of the major traders of the meat. |
| 403 | Dried meat but in small numbers |
| 405 | The fishermen catch eagle mantas to eat them. |
| 417 | As above, whole rays are sometimes for sale in the Jeddah fish market. |
| 419 | Fish markets in muscat/Dubai seen carried by fisherman on beach |
| 419 | rays are found in the fish market. so are baby sharks and every kind of fish they catch. funny enough, no turtles though. |
